# Expired Patents

**Published:** 1907-05

**Authors:** 


EXPIRED PATENTS.

March 25 ,1907.

424050. Artificial Tooth. G. L. Curtis, Chicago, Ill.

April 1, 1907.

424790. Dental Matrix Retainer. J. W. Ivory, Phila. Pa.
424924. Dental Bridge. G. L. Curtis, Syracuse, N. Y.

April 8, 1907.

425067. Mechanism for Separating Teeth. J. N. Farrar, New York, N. Y.
425344. Dental Plugger. F. C. Ries, Macon, Ga.

April 15, 1907.

425650. Dental Tooth. B. J. Ring, Paris, France.
425654. Truck for Dental Chairs. B. S. Brown, Boston, Mass.
425897. Dental Polishing Dish. W. N. Morrison, St Louis, Mo.

The above lists of patents are furnished by Davis & Davis, Solicitors of American and Foreign Patents, Washington, D. C., and St. Paul Building, New York City.

